# Integration of Technology in Medical Education on Primary Care During the COVID-19 Pandemic: Students’ Viewpoint

**DOI:** 10.2196/22926

**Published:** 2020-11-18

**Authors:** Nadine Paul, Sae Kohara, Gursharan Kaur Khera, Ramith Gunawardena

**Affiliations:** 1 King's College London London United Kingdom

**Keywords:** clinical education, curriculum development, personal characteristics, physician/patient relationship, professional development, education, medical student, telemedicine, simulation, COVID-19

## Abstract

The COVID-19 pandemic has forced medical schools and clinicians to transition swiftly to working online, where possible. During this time, final-year medical students at King’s College London, England, have received some of their general practice teachings in the form of virtual tutor groups. The predominant feature of such groups is online patient simulations, which provide students a valuable experience to help gain insight into current clinical practice amid the pandemic and inform how their practices as incoming junior doctors would continue. Even in the absence of face-to-face teaching and clinical placements, students have been able to hone their medical knowledge and soft skills through these virtual, simulated consultations. They have been exposed to a new consultation style while in a safe and collaborative learning space. Here, we explore how medical students have benefited from these virtual tutor groups and how similar small-group online teaching opportunities can add value to the medical curriculum in the future.

## Introduction

The upward trend in the use of digital health consultation applications has been greatly accelerated by the current COVID-19 pandemic [1]. This has compelled general practitioners, in particular, to rapidly shift from face-to-face consultations to telephone or video interactions wherever possible [2]. In many cases, this transition has been swift, and many health care professionals have experienced a steep learning curve. As final-year medical students at King’s College London, England, our general practice teaching has also transitioned to a web-based format. In particular, virtual tutor groups (VTGs) have been introduced as a key component of the new online learning format. VTGs consist of small-group teaching sessions organized weekly that are supervised by an experienced general practice tutor. Students meet via a web-based video conferencing application such as Microsoft Teams (Microsoft Corporation) for interactive scenario- or simulation-based teaching. Students are required to manage patient cases via virtual consultations in a manner similar to how many general practitioners are currently practicing; these consultations are followed by feedback, discussion, and teaching. We have been using the VTG format for several months, and our positive experiences have given us a strong reason to believe that the benefit of these online group sessions span far beyond continuity in teaching. VTGs have given us the opportunity to practice consultation skills within a supervised and supportive environment while abiding social distancing guidelines. As we face a potential resurgence of COVID-19, the value of these skills is now more pertinent than ever.

## Using Technology to Supplement Clinical Experience

In addition to bridging the gaps in teaching in primary care, VTGs have become an extremely useful and novel format for engagement during our time away from clinical placements. Patients are not physically present during the consultation; therefore, it becomes essential to perform a structured assessment of how unwell a patient is, merely through an audio or video-based interaction. We are encouraged to think laterally about how to perform a physical examination and use equipment that the patient may already have in their homes to aid diagnosis, such as home-based blood pressure monitors, peak flow meters, and oxygen saturation probes. Furthermore, we are often challenged to optimize our verbal communication in order to effectively lead a virtual consultation in the absence of physical interaction and non-verbal cues. This prompts reflection and, subsequently, adaptation to these new consultation styles. During all consultations, we are required to cover certain essential components such as addressing the patients’ concerns, sharing a management plan, and providing adequate safety nets. 

## Key Challenges

As the prevalence of online consultations grows, the challenges associated with this style of practice also become apparent. Shaw et al [3] dispelled a common trope that despite technical difficulties, clinicians and patients can work collaboratively to find means to overcome them. Guidance is provided to aid clinicians to be able to evaluate whether remote patient examination is suitable and appropriate [4]. As students, we have found it challenging to determine whether inviting a patient to examine them in person would actually alter our management plan or whether they could be directed by virtual consultation to either be managed at home with safety netting or referred directly to secondary care; this challenge has been further compounded by our limited clinical exposure in recent times. Furthermore, an issue that we, as students, discovered was the lack of guidance for respecting patient exposure during virtual examinations and assessments; this was particularly true with regard to data protection and patient confidentiality. In order to safeguard patient privacy and maintain a positive physician-patient relationship, medical professionals must utilize medical software and video-conferencing applications with appropriate integrated security systems. The importance of data protection and privacy should be highlighted as part of the curriculum when guiding students through telemedicine training. This has led to productive discussions for different cases among our groups, with guidance from our general practice tutors.

## Soft Skills and Teleconsultations

With reference to soft skills, we have received positive feedback from patients participating in our online simulated consultations. These patients mentioned that students consulting them expressed a great amount of empathy and were clear in their communication, despite the physical and technological barriers. We have found that reflecting, and subsequently, adapting our communication styles to suit telephone and video consultations proves to be a valuable learning experience, and one which we will draw upon often in the future. For example, over video consultations, verbal cues can be disruptive to the conversation; hence, the emphasis is on the importance of communicative facial expressions and body language. In contrast, means of non-verbal communication such as nodding or other silent methods of active listening are futile in telephone conversations. In the latter case, we must place greater emphasis on verbal acknowledgement without affecting the flow of conversation.

## Group Learning

In addition to equipping students with essential skills in virtual consultations, we have found that VTGs create a strong sense of community among peers. The close-knit and collaborative learning environment has been especially welcomed in a time where didactic online teaching has largely been an isolating experience. Apart from conducting the simulated consultations ourselves, it has been an enriching experience to observe our peers navigate through complex scenarios. Giving and receiving feedback has also been confidence-inducing. The VTGs have allowed us the opportunity to develop our communication skills as well as share and integrate varied communication styles among us. This has allowed us to feel well prepared and enthusiastic for our return to the clinical environment, albeit somewhat virtually.

## Conclusions

The role of technology in health care is undoubtedly expanding at a rapid pace, and this is especially true in the COVID-19 era, where noncontact solutions to health care needs have become essential. As final-year medical students, we believe that it is crucial that we are equipped to adapt to different formats of remote working and to address any associated challenges that may present in the future. There have been several technological adaptations to global medical curricula during the past months, such as the transition of physical lectures to web-based formats; however, many other aspects of medical teaching have been paused until face-to-face teaching can safely be resumed. We believe that by integrating simulated remote consultations through VTGs, students can continue to develop their communication skills and clinical acumen, and this should be considered as a permanent inclusion in the post–COVID-19 medical curricula.

## Abbreviations

VTG: virtual tutor groups
